# Roles of GSK-3 and β-Catenin in Antiviral Innate Immune Sensing of Nucleic Acids

**DOI:** 10.3390/cells9040897

**Published:** 2020-04-07

**Authors:** Alexandre Marineau, Kashif Aziz Khan, Marc J. Servant

**Affiliations:** 1 Faculty of Pharmacy, Université de Montréal, Montréal, QC H3C3J7, Canada; alexandre.marineau@gmail.com; 2 Department of Biology, York University, Toronto, ON M3J1P3, Canada; kakhan@yorku.ca; 3 Réseau Québécois de Recherche sur les Médicaments (RQRM), Montréal, QC H3T1C5, Canada

**Keywords:** GSK-3, β-catenin, innate antiviral immunity, toll-like receptors (TLR), RIG-like receptors (RLR), cGAS, TBK1, type I IFN response

## Abstract

The rapid activation of the type I interferon (IFN) antiviral innate immune response relies on ubiquitously expressed RNA and DNA sensors. Once engaged, these nucleotide-sensing receptors use distinct signaling modules for the rapid and robust activation of mitogen-activated protein kinases (MAPKs), the IκB kinase (IKK) complex, and the IKK-related kinases IKKε and TANK-binding kinase 1 (TBK1), leading to the subsequent activation of the activator protein 1 (AP1), nuclear factor-kappa B (NF-κB), and IFN regulatory factor 3 (IRF3) transcription factors, respectively. They, in turn, induce immunomodulatory genes, allowing for a rapid antiviral cellular response. Unlike the MAPKs, the IKK complex and the IKK-related kinases, ubiquitously expressed glycogen synthase kinase 3 (GSK-3) α and β isoforms are active in unstimulated resting cells and are involved in the constitutive turnover of β-catenin, a transcriptional coactivator involved in cell proliferation, differentiation, and lineage commitment. Interestingly, studies have demonstrated the regulatory roles of both GSK-3 and β-catenin in type I IFN antiviral innate immune response, particularly affecting the activation of IRF3. In this review, we summarize current knowledge on the mechanisms by which GSK-3 and β-catenin control the antiviral innate immune response to RNA and DNA virus infections.

## 1. Introduction

In mammals, glycogen synthase kinase-3 (GSK-3) refers to two paralogous genes, *GSK3A* on chromosome 19 and *GSK3B* on chromosome 3, that generate two related protein isoforms, GSK-3α and GSK-3β [1,2]. These serine/threonine protein kinases are expressed ubiquitously in most cell types. Structurally, the kinase domain of both GSK-3 isoforms share 85% and 98% similarities in their nucleic acid sequence and amino acid identity, respectively. However, both proteins share only 36% similarity in the last 76 amino acids of their C-terminal regions [3]. Through the phosphorylation of over 40 validated substrate proteins (hundreds of other potential GSK-3 substrates still need to be validated) [4,5], they control multiple cellular functions including glucose homeostasis, growth, differentiation, cell mobility, migration, apoptosis, and inflammatory response. Today, several GSK-3 pharmacological inhibitors exist that aim at targeting the dysregulation of these isoforms that are linked in the initiation and progression of several human diseases [6,7,8,9,10,11].

Studying the contributions of GSK-3 in the integration of many extracellular cues leading the optimal or deregulated cellular responses has come with many technical and conceptual challenges. Likely related to their high overall homology, both isoforms share redundant roles [12,13,14,15,16]. However, they also play unique roles in many physiological and pathological conditions including cardiac pathogenesis, atherosclerosis, hepatic glycogen metabolism, glucose homeostasis, fatty acid accumulation, cell survival, aging, male fertility, and inflammation [17,18,19,20,21,22,23,24,25,26]. Interestingly and in opposition to the vast majority of protein kinases, GSK-3 isoforms are active in unstimulated resting cells, and the efficiency of GSK-3 phosphotransferase activity is tightly regulated by certain cellular events. For instance, upon growth factors stimulation, they become inactive through phosphorylation by serine/threonine kinase/protein kinase B (AKT) and p90RSK at residue Ser9 on GSK-3β and Ser21 on GSK-3α. Nevertheless, the phosphorylation of Tyr279 in GSK-3α and Tyr216 in GSK-3β (located in the T-loop) increases the phosphotransferase activity towards their substrates. Additional post-translational modifications have been described, especially for the GSK-3β isoform. The p38 mitogen-activated protein kinase (MAPK) induces the inactivation of nuclear GSK-3β upon its phosphorylation at Ser389 [27]. Mono ADP-ribosylation by ARTD10 also decreases its activity, whereas the N-terminal citrullination by PAD4 promotes its nuclear accumulation [28,29]. Moreover, GSK-3β may also act as a scaffold protein, independently of its catalytic activity. As detailed below, in response to specific inflammatory triggers, GSK-3β interacts with the E3 ubiquitin ligase tumor necrosis factor (TNF) receptor-associated factor 6 (TRAF6) and undergoes K63-linked polyubiquitination at Lys183, a modification required for the production of proinflammatory cytokines [30]. Both isoforms play various roles in several signaling pathways including the Wnt, Ras/MAPK, cyclic AMP, transforming growth factor-β/activin, Notch, Hedgehog, phosphatidylinositol-3 kinase (PI3K), jun kinase/stress-activated protein kinase (JNK/SAPK), nuclear factor-kappa B (NF-κB), and the Janus kinase/signal transducer and activator of transcription (JAK/STAT) pathways. However, one of the most characterized substrates of the GSK-3 isoforms is the transcriptional co-activator β-catenin involved in the Wnt/β-catenin pathway [6,31,32].

**Figure 1:**
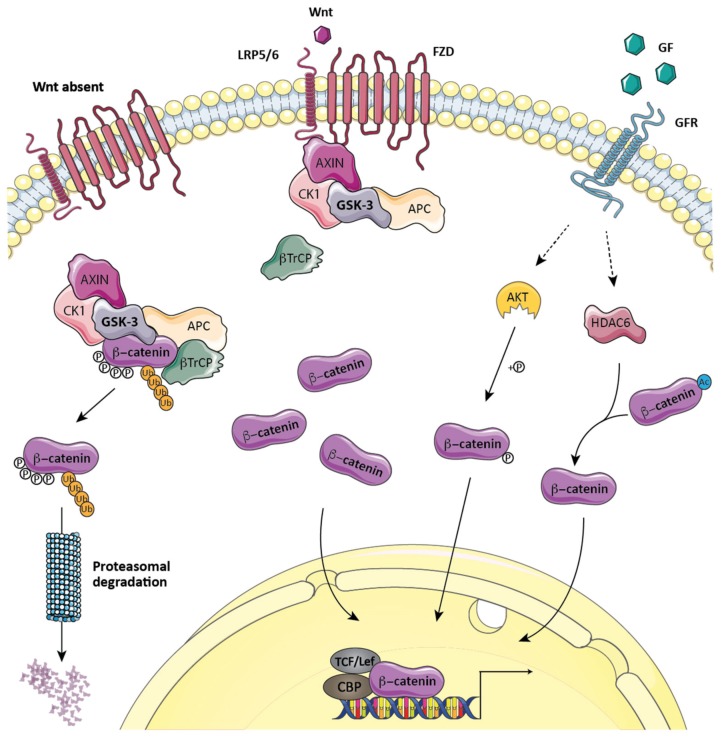
Wnt/β-catenin signaling. Whereas the vast majority of β-catenins function as adapter molecules at the cell membrane, linking cadherin receptors to the actin cytoskeleton (not shown), a small cytoplasmic pool of β-catenins plays key roles in transcriptional events. In the absence of Wnt ligands, the level of β-catenin in cytoplasm is kept low by a process involving the ubiquitin-proteasome system that employs a multiprotein destruction complex containing glycogen synthase kinase (GSK)-3α/β, casein kinase 1 (CK1), axin, adenomatous polyposis coli (APC), protein phosphatase 2A (PP2A) (not shown), and the E3-ubiquitin ligase beta-transducin repeats-containing proteins (β-TrCP). The engagement of Wnt ligands to frizzled/LRP receptors results in the destabilization of the destruction complex (the release of β-TrCP), thus allowing for the cytoplasmic accumulation of neo-synthetized β-catenin, followed by its nuclear translocation and binding to lymphoid enhancer factor/T-cell factor (LEF/TCF). Along with CBP/p300, they induce a gene network involved in cell proliferation, differentiation, and lineage commitment. Another mechanism of β-catenin nuclear translocation is the growth factor (GF) receptor (GFR)-mediated, AKT-dependent phosphorylation of β-catenin at Ser552 or its deacetylation by HDAC6 at Lys49. The model was created using Servier Medical Art templates (www.servier.com) licensed under a CC BY 3.0 license (https://creativecommons.org/licenses/by/3.0/).

Wnt ligands play a vital role in the maintenance of tissue homeostasis by regulating cell proliferation, differentiation, migration, survival, genetic stability and in upholding adult stem cells in a pluripotent state [33]. Though there are multiple Wnt genes in animal genomes, they all share the same biochemical signal transduction mechanism: controlling the expression level of β-catenin, the key effector of the canonical Wnt/β-catenin signaling pathway. When Wnt ligands do not engage frizzled/LRP receptors, the level of the cytoplasmic pool of β-catenin is kept low by degradation via a multiprotein destruction complex containing GSK-3α/β, casein kinase 1 (CK1), axin, adenomatous polyposis coli (APC), protein phosphatase 2A (PP2A), and the E3-ubiquitin ligase beta-transducin repeats-containing proteins (β-TrCPs) [34]. The scaffolding of β-catenin, GSK-3, and CK1 by axin allows for the sequential phosphorylation of β-catenin occurring at regularly spaced N-terminal Ser/Thr residues; Ser45 is phosphorylated by CK1, which primes subsequent GSK-3α/β-mediated phosphorylation at residues Thr41, Ser37, and Ser33. The created so-called phosphodegron motif (p-Ser33 and p-Ser37) provides a binding site for β-TrCP, resulting in the proteosomal degradation of β-catenin [35]. Upon the binding of Wnt ligands to their cognate receptors, the destabilization of the destruction complex allows for the rapid accumulation of neo-synthetized β-catenin, as well as its nuclear translocation and binding to lymphoid enhancer factor/T-cell factor (LEF/TCF) and CBP/p300 [36], where it acts as a transcriptional coactivator that controls the expression of a gene network involved in cell proliferation, differentiation, and lineage commitment [33]. Interestingly, the nuclear accumulation of β-catenin has been also observed following growth factor (GF) receptor (GFR)-mediated, AKT-dependent β-catenin phosphorylation at Ser552 [37,38] and its deacetylation by HDAC6 at Lys49 [39], modifications involved in its dissociation from cell–cell contacts and preventing its phosphorylation at Ser45, respectively (Figure 1). 

Wnt signaling regulates the immunological response to pathogenic infections and has already been covered in detail elsewhere [40,41,42,43,44]. However, no clear picture has emerged regarding the contributions of the Wnt ligands in the antiviral response to different classes of viruses. Nonetheless, intracellular signal transduction models were recently proposed. In these models, GSK-3 and β-catenin, alone or in conjunction, influence the type I interferon (IFN) antiviral response following nucleic acid sensors activation.

## 2. Antiviral Innate Immunity

**Figure 2:**
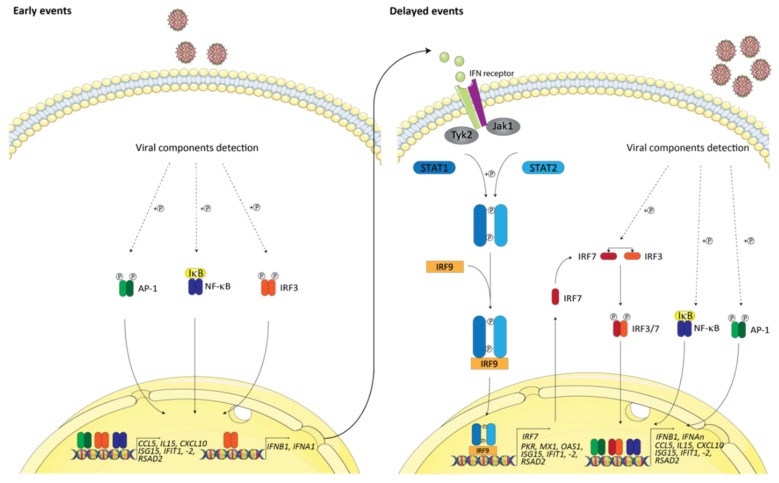
Early and delayed events in interferon signaling. The recognition of viruses by cells triggers the rapid activation of multiple latent transcription factors, namely nuclear factor-kappa B (NF-κB), activator protein 1 (AP-1) (ATF-2/c-Jun), and interferon regulatory factors (IRFs) via the IκB kinase (IKK) complex, mitogen-activated protein kinases (MAPKs), and IKK-related kinases, respectively (not shown). These transcription factors, in turn, directly activate a set of immunomodulatory genes including interferon (IFN)-stimulated genes (ISGs) and cytokines (RANTES, IP-10, and type I IFN). Once secreted, type I IFNs (IFN-α and IFN-β) act in a paracrine and autocrine fashion to amplify the initial production of type I IFN and to induce other ISGs. In fact, upon binding to interferon-α/β receptor (IFNAR), type I IFN leads the activation of Tyk2 and Jak1, which then phosphorylate signal transducer and activator of transcription 1 (STAT1) and STAT2, leading to their dimerization and to the recruitment of IRF9 to form the ISGF3 complex. The binding of this complex to interferon-stimulated response elements (ISRE) found in the promoter of a multitude of ISGs, including IRF-7, thus results in the amplification of antiviral response. IRF3/IRF7 heterodimers, in conjunction with AP-1 and NF-κB, allow for the production of type 1 interferons and ISGs, resulting in growth inhibition, apoptosis, and impaired viral gene expression and replication. The AP-1 complex depicted here is composed of the ATF-2/c-Jun heterodimers. Following the phosphorylation of ATF2 by the MAPK p38/JNK (jun kinase) in the cytoplasm and the translocation of these activated protein kinases into the nucleus where they also target c-Jun, the formation of an active AP-1 complex, in fact, occurs in the nucleus following virus infection (for the simplicity of figure, the activating kinases are not shown). The model was created using Servier Medical Art templates (www.servier.com) licensed under a CC BY 3.0 license (https://creativecommons.org/licenses/by/3.0/).

Viral infection triggers the rapid activation of NF-κB, activator protein 1 (AP1), and the interferon regulatory factor (IRF) transcription factors, of which IRF3 and 7 are the most characterized. These transcription factors, in turn, directly activate a set of immunomodulatory genes including IFN-stimulated genes (ISGs) such as those for viperin (*RSAD2*), ISG56 (*IFIT1*), and ISG54 (*IFIT2*), as well as those encoding for proinflammatory chemokines (RANTES (*CCL5*), IP-10 (*CXCL10*)), and the type I IFN antiviral cytokines (IFNα/β) [45]. Notably, the transcription of the *IFNB1* gene requires the coordinated actions of IRF3, NF-κB, and AP1 [46,47], whereas the 13 genes encoding for the IFNα subtypes use IRF7 transcription factor [48,49]. In specialized immune cells (e.g., dendritic cells and macrophages), IRF7 is constitutively expressed and can also participate in the induction of IFNβ. In the vast majority of cells, however, IRF7 is only minimally expressed and instead acts as an ISG in the well-accepted two-step amplification model shown in Figure 2 [50,51,52]. Once produced, type I IFNs control gene expression following binding to the cell surface type I IFN α/β receptors, thus resulting in the activation of the JAK/STAT signaling pathway and the formation of the interferon-stimulated gene factor 3 (ISGF3) complex. This complex then binds to interferon-stimulated response elements (ISRE) found in the promotors of numerous ISGs, resulting in the amplification of the antiviral response [45]. Since type I IFNs and ISGs act as endogenous potent antiviral agents and are powerful immunological modulators, a clear understanding of the mechanisms of their expression in infected cells is required to identify novel cellular targets for future antiviral or autoimmune therapies.

## 3. Viral Nucleic Acids Recognition

In the past few years, seminal discoveries in the field of type I IFN antiviral response have significantly increased our understanding of how immune and non-immune cells recognize and respond to viruses. Several members of the evolutionarily conserved receptors family, termed pattern-recognition receptors (PRRs), are specially encoded to detect and react with conserved essential molecular determinants found in microorganisms called pathogen-associated molecular patterns (PAMPs). Once engaged by the latter, activated PRRs rapidly induce an innate immune response that leads to complex inflammatory and immunoregulatory reactions that protect the host from the ongoing infection. Whereas the vast majority of PRRs rely on the activation of the NF-κB and AP1 transcription factors for the establishment of a potent inflammatory response, the IRF3/7-dependent production of antiviral and immunoregulatory IFNα/β mostly depends on nucleotide-sensing PRRs. These receptors/sensors responsible for detecting DNA and RNA viruses include the cytoplasmic DNA sensors (RNA polymerase III, cyclic GMP–AMP synthase (cGAS), and IFI16); the endolysosomal toll-like receptors (TLRs) 3, 7, 8, and 9; and the cytoplasmic RIG-I-like receptors (RLRs) RIG-I, MDA5 and LGP2 (reviewed in [53,54,55]). 

## 4. Cytosolic RNA Sensors

**Figure 3:**
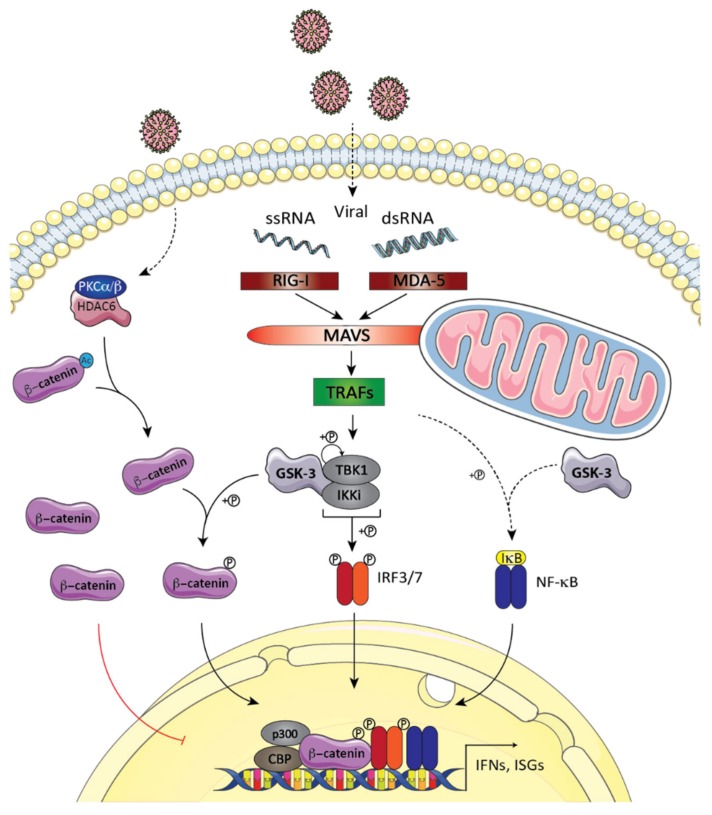
Fine-tuning the RLR-induced type I IFN response by GSK-3 and β-catenin. The recognition of double-stranded RNA (dsRNA) molecules and 5′triphosphate single-stranded RNA (ssRNA) by MDA5 and RIG-I, respectively, induces the polymerization of the mitochondrial adaptor protein MAVS (mitochondrial antiviral signaling). The recruitment of tumor necrosis factor (TNF) receptor-associated factors (TRAFs) to polymerized MAVS results in the activation of the constitutively expressed protein kinase TANK-binding kinase 1 (TBK1) and its inducible homologue IKKi (or IKKε). GSK-3 acts as a scaffolding entity that allows for the oligomerization and activation, of TBK1, leading to the phosphorylation and activation of transcription factors like IRF3. The activation of IRF3 following viral infection involves multiple phosphorylation events at its C-terminal, which are followed by its dimerization, nuclear translocation, and interaction with co-activators CBP and p300. β-catenin deacetylated by HDAC6 and phosphorylated by GSK-3 acts as an IRF3 co-activator by interaction with holocomplex formed by IRF3 and CBP/p300. GSK-3β expression is also necessary for proper IκB phosphorylation and subsequent NF-κB activation. On the other hand, the accumulation of unphosphorylated ‘‘active’’ β-catenin appears to limit the antiviral response. Arrows represent activation, whereas blocking arrows signify inhibition. The model was created using Servier Medical Art templates (www.servier.com) licensed under a CC BY 3.0 license (https://creativecommons.org/licenses/by/3.0/).

RIG-I and MDA5 are related DExD/H box RNA helicases and are among the most critical cytoplasmic sensors for viral RNA and trigger the RLR signaling pathway [56,57,58,59]. Once activated by double-stranded RNA (dsRNA) molecules and 5′triphosphate single-stranded RNA (ssRNA), MDA5 and RIG-I are recruited and induce the polymerization of the mitochondrial adaptor protein known as mitochondrial antiviral signaling (MAVS; also known as IPS-1, Cardif, and VISA) [60,61,62,63,64]. Polymerized MAVS then recruits TRAF 2, 3, 5, and 6 E3 ubiquitin ligases, culminating in the activation of the constitutively expressed protein kinase TANK-binding kinase 1 (TBK1) (Figure 3) [65,66]. TBK1 and its inducible homolog IKKi (or IKKε) are known as the IκB kinase (IKK)-related kinases [67]. Under the condition of overexpression, these kinases were first characterized as NF-κB inducers before being recognized as the molecular entities that phosphorylate and activate the IRF3 and IRF7 transcription factors [68,69]. The activation of IRF3 has been well characterized. Following multiple phosphorylation events at its C-terminal, IRF3 dimerizes and translocates into the nucleus, where it interacts with the transcriptional co-activators CBP, p300 and PCAF [68,69,70,71,72], leading to the robust induction of an antiviral gene program [73]. New signal transduction networks have been proposed to explain the cross talk that exists between GSK-3 and/or β-catenin and effectors of the RLR pathway. Due to the paucity of these studies, the working models have sometimes been in contradiction. However, pictures are emerging where (1) GSK-3 operates at the level of β-catenin [74] and/or TBK1 [75,76]; and (2) β-catenin acts via the classical holocomplex formed by IRF3 and CBP/p300 [74,77,78,79,80,81] or TCF [82] to regulate the production of type I IFNs (Figure 3). 

Though recently demonstrated to be involved in the TCF-dependent basal production of IFNβ [82], the first study implying a role of β-catenin in virus-induced *IFNB1* transcription came with the discovery of the role of LRRFIP1, a β-catenin and RNA-binding protein acting in parallel to the RLR-TBK1-IRF3 pathway, in the production of type I IFN [78]. Once engaged by nucleic acids, LRRFIP1 is found in immunocomplexes containing β-catenin, where it enables its phosphorylation at Ser552 and accumulation in the nucleus. Once in this environment, β-catenin interacts with IRF3, facilitating the recruitment of p300 and the acetylation of the *IFNB1* promoter. Accordingly, upon vesicular stomatitis virus (VSV) challenge, cultured cells and mice deficient in β-catenin produced less IFNβ and, subsequently, reduced antiviral innate immune responses [78]. Later on, other studies supported the induction of a β-catenin-IRF3-p300/CBP complex upon infection with Sendai virus (SeV) and influenza A virus H1N1 [79,80,83] that acts in parallel to the RLR-TBK1-IRF3 pathway to fine-tune the antiviral innate immune response. Deacetylation at Lys49 by calcium-sensitive PKCα/β-activated HDAC6 is required for the nuclear translocation of β-catenin and its association with IRF3 and CBP/p300 [80]. The contribution of the phosphoacceptor site Ser552 in the HDAC6-mediated nuclear accumulation of β-catenin was not verified in these studies.

Though previously shown to be required in influenza virus entry steps [84], GSK-3 is also essential for the induction of antiviral immune response and is, in fact, the target of RNA viruses. One example is the NS5A protein of the hepatitis C virus (HCV) that has been reported to inhibit GSK-3 and lead to the stabilization of unphosphorylated β-catenin [85,86]. This specific form of β-catenin, not phosphorylated on Ser33/37 and Thr41, is the functionally “active” form found in cell–cell adhesion and the canonical Wnt pathway [35,87]. It would be unlikely that this is the form present in the holocomplex formed by IRF3 and CBP/p300 described above. We and others have observed that when this form accumulates in response to the use of GSK-3 inhibitors, in the presence of Wnt ligands, or through β-catenin overexpression, it negatively affects the type I IFN response in cells following RLR stimulation [88,89,90]. However, other groups have reported the opposite in response to RNA viruses, including influenza A virus, human immunodeficiency virus (HIV), bovine parainfluenza virus type 3, and Rift Valley fever virus [40,81,82,91,92].

These discrepancies need to be addressed and could be related to the use of different types of RNA viruses, ectopic overexpression conditions, and the use of transformed cell lines and non-selective GSK-3 inhibitors such as lithium [93]. Moreover, neither the presence nor the role of the “inactive” GSK3-phosphorylated form of β-catenin have been addressed in most studies. It is worth noting that the phosphorylation of β-catenin at Thr41, Ser37, and Ser33 does not inevitably lead to its degradation but may have important regulatory functions [87,94]. Indeed, N-terminally phosphorylated β-catenin has essential roles in microtubule organization at the centrosomes [95,96], cell adhesion, and migration [97,98], maintaining neuroepithelial integrity in developing midbrain [99], promoting neuronal excitability [100], and enhancing the transcriptional activity of the β-catenin/TCF4 complex [101]. It could be proposed that the “active” non-phosphorylated form of β-catenin is required for the replication of viral constituents [102], as well as constitutive-basal IFNβ production [82]. Nevertheless, the GSK-3-modified version of β-catenin would be required for an optimal antiviral immune response. In fact, upon SeV infection, we recently documented the presence of the “inactive” GSK-3 phosphorylated form of β-catenin within the *IFIT1* promoter in association with the IRF3 holocomplex in primary, immortalized, and transformed cell lines [74], arguing that the phosphotransferase activity of GSK-3 is required for an optimal type I IFN antiviral response. In support of this, we showed that the deletion of the phosphodegron motif of β-catenin decreases the DNA binding activity of IRF3 and the antiviral innate immune response following SeV and VSV infections. The phosphorylation of glycogen synthase at Ser641 following SeV infection and the increase in the phosphotransferase activity of GSK-3 observed through immunocomplex in vitro kinase assays have been additional observations demonstrating an increase in the catalytic activity of GSK-3 towards its substrates [74]. Interestingly, in addition to SeV and VSV, the activation of GSK-3 has previously been reported during infection with coxsackievirus, an RNA virus [103], as well as in response to the HIV-1 Tat protein [104]. In line with these results, influenza A virus infection results in the downregulation of the Wnt pathway in alveolar epithelial cells [105], and a phosphoproteomic analysis of infected samples showed an enrichment of substrates of GSK-3 [106]. At the moment, it is still unknown how GSK-3, which is constitutively active in resting cells, engages β-catenin following virus infection, but thus could involve TBK1, as discussed below. To our knowledge, only one report so far has proposed a negative role of catalytically active GSK-3 in IRF3-dependent gene transcription using non-selective GSK-3 inhibitor lithium [107]. Paradoxically, lithium treatment may also result in the hyperphosphorylation of β-catenin on Ser33 and Ser37 [108], a situation that could actually induce the production of type I IFN, explaining its antiviral effects [82].

The roles of GSK-3 in RLR signaling events were first documented by Lei et al. [75]. Based on silencing approaches in 293T cells and rescue experiments with both GSK-3 isoforms into Gsk-3β^-/-^ mouse embryonic fibroblasts (MEFs), they concluded that GSK-3β, but not GSK-3α, was involved in IRF3 activation and the induction of type I IFN upon SeV infection. They further observed that the reconstitution of the RLR pathway also occurred with an ATP-binding deficient mutant of GSK-3β, and that it was present in TBK1 immunocomplexes derived from 293T cells infected with SeV. They proposed that GSK-3β acts as a scaffolding entity in the RLR pathway, operating downstream of RIG-I and MAVS and allowing TBK1 to self-associate and transautophosphorylate at Ser172, a residue that has been previously shown to be present in the activation loop and to be essential for catalytic activity [109]. Interestingly, Gsk-3β^-/-^ MEFs also show a significant defect in IκBα phosphorylation and degradation upon SeV infection, thus implying a role of GSK-3β in the activation of the IKK complex through an unknown mechanism [75] (Figure 3). This seems to work in a distinct manner compared to TLR2/4/5/9- and TNFα-induced NF-κB activation, in which the loss of GSK-3β does not affect the early activation steps (i.e., IκBα degradation and nuclear translocation) [22,110]. Though convincing in many ways, one major caveat of Lei et al.’s study was the presence of the endogenous GSK-3α isoform in reconstituted assays. 

Thus, to further clarify the role of GSK-3α and GSK-3β in antiviral signaling, we used an allelic series of undifferentiated mouse ES cells lacking GSK-3 isoforms (WT, GSK-3α^-/-^, GSK-3β^-/-^, or GSK-3α/β DKO) or GSK-3α/β DKO ES cells reconstituted with the catalytically inactive versions of GSK-3 isoforms, as well as RNAi approach targeting GSK-3α in Gsk-3β^-/-^ MEFs. These cellular models showed not only that both catalytically active isoforms are involved in RLR-induced antiviral IRF3 gene program but also that neither the phosphorylation of TBK1 at Ser172 nor the phosphorylation and the nuclear translocation of IRF3 was affected by their absence [74]. In fact, our observations allowed us to propose a model where virally-activated GSK-3 isoforms selectively engage β-catenin for its phosphorylation, which in turn is recruited to the IRF3 holocomplex for its optimal DNA binding and the induction of antiviral genes (Figure 3). As another group also recently documented the presence of endogenous TBK1 in ectopically expressed GSK-3β immunocomplexes [76], one could argue that it is within this complex that TBK1 controls the ability of constitutively active GSK-3 isoforms to engage β-catenin.

## 5. Cytosolic DNA Sensors

**Figure 4:**
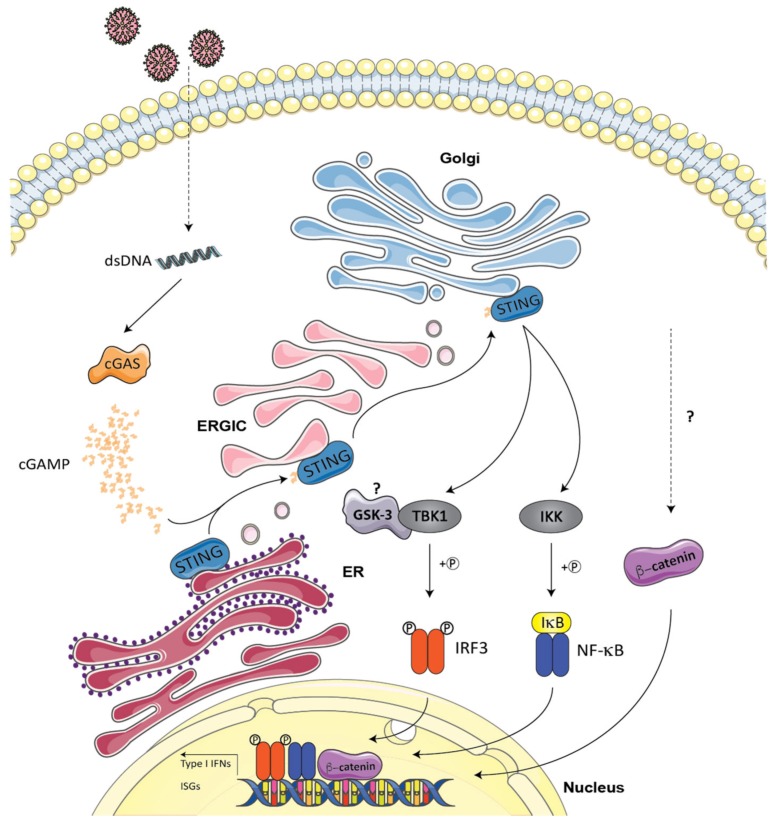
GSK-3/β-catenin in sensing of cytosolic DNA by the cyclic GMP–AMP synthase (cGAS)/STING pathway. Upon infection by DNA viruses, several DNA sensors molecules show overlapping abilities in their capacity to induce a type I IFN antiviral response, but cGAS is the most characterized. Upon binding with DNA molecules, it rapidly produces a second messenger cyclic GMP-AMP (cGAMP) that activates the endoplasmic reticulum (ER)–resident adaptor protein STING. In a complex with cGAMP, STING translocates from the ER to the ERGIC/Golgi to activate TBK1-IRF3 and NF-κB, resulting in robust type I IFN induction and inflammatory cytokine production. Limited information is available on the cross talk between the GSK-3/β-catenin and cGas/STING pathways. It may happen through either the recruitment of GSK-3 to TBK1 or through the binding of p-β-catenin Ser552 with the IRF3 promoter in association with TCF4 for the induction of type I IFN. The model was created using Servier Medical Art templates (www.servier.com) licensed under a CC BY 3.0 license (https://creativecommons.org/licenses/by/3.0/).

Upon infection by DNA viruses, several DNA sensors molecules show overlapping abilities in their capacity to induce a type I IFN antiviral response. AT-rich dsDNA sequences can be recognized and transcribed by RNA polymerase III into 5′-triphosphate double-stranded RNA (5′-ppp-dsRNA), which in turn activates the RIG-I–MAVS pathway described above [111,112]. However, cGAS is the most characterized [113] (see [53,54] for reviews). Once engaged by DNA molecules, it rapidly leads to the production of cyclic GMP-AMP (cGAMP) [114,115], which acts as a second messenger molecule that activates the endoplasmic reticulum (ER)-resident adaptor protein STING [116,117]. In complex with cGAMP, STING translocates from the ER to the ERGIC/Golgi to activate TBK1-IRF3 and NF-κB, resulting in robust type I IFN induction and inflammatory cytokine production [118] (Figure 4). The detection of DNA by other DNA sensors like IFI16 and AIM2 in the nucleus and cytoplasm, respectively, activates the inflammasome via the recruitment of ASC and caspase-1, leading to the proteolytic cleavage of pro-IL1β and pro-IL18 (see [54] for review). At the moment, the cross talk that may exist between GSK-3/β-catenin pathways and the effectors described above were documented at the level of the recruitment of GSK-3 to TBK1 in herpes simplex virus (HSV-1)-infected cells [76]. Recently, the requirement of β-catenin in the cGAS/STING mediated activation of the IFN pathway was shown in a reporter assay [119]. This could likely happen through the binding of p-β-catenin Ser552 with IRF3 promoter in association with TCF4, as reported following STING activation in *Toxoplasma gondii* infection [120]. It is interesting to note, however, that viral interference inhibiting the functions of both GSK-3 and β-catenin has been observed with many DNA viruses-encoded proteins including the hepatitis B virus (HBV) X protein [121], the Epstein–Barr virus (EBV) LMP2A [122,123], the latency-associated nuclear antigen of Kaposi’s sarcoma-associated herpesvirus (KSHV) [124], and the US3 protein from HSV-1 [119]. In addition to viral interference antagonizing the GSK-3/β-catenin pathways, other DNA viruses, such as human cytomegalovirus, use GSK-3 at their advantage to induce the degradation of SPOC1 (survival time-associated PHD (plant homeodomain) finger protein in ovarian cancer 1), a transcriptional coregulator involved in the inhibition of viral immediate-early (IE) gene expression [125].

## 6. Endolysosomal DNA and RNA Sensors

**Figure 5:**
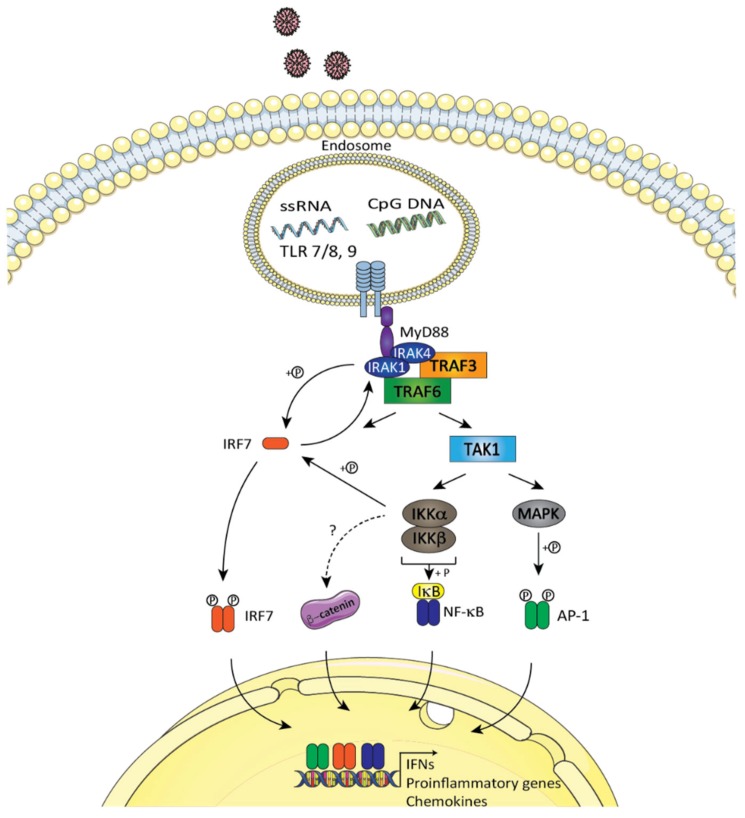
GSK-3/β-catenin in endosomal nucleic acid sensing by toll-like receptor (TLR)7/8 and TLR9. The engagement of TLR7/8 by ssRNA and TLR9 by CpG DNA results in the activation of adapter protein MyD88 to recruit IRAK family kinases in complex with the E3 ubiquitin ligases TRAF3 and TRAF6. Transcription factor IRF7 is recruited to this macromolecular complex followed by its phosphorylation by the protein kinase IKKα and translocation to the nucleus where it induces the expression of type I IFN. TRAF6 also positively regulates NF-κB and activator protein 1 (AP1_ transcription factors through MAPKKK TAK1 and the IKK complex, thus leading to the initiation of a pro-inflammatory gene activation. The model was created using Servier Medical Art templates (www.servier.com) licensed under a CC BY 3.0 license (https://creativecommons.org/licenses/by/3.0/).

As opposed to the constitutive and ubiquitous nature of cytosolic nucleic acid sensors, endolysosomal TLRs are preferentially expressed in myeloid cells, such as dendritic cells and macrophages [45]. Following their internalization by the host, virus particles traffic to the endosomal compartment, where viral products are exposed to TLR3/7/8/9. For instance, infection with influenza A virus, an RNA virus, produces dsRNA molecules that are recognized by TLR3. DNA viruses such as vaccinia virus, adenovirus, and herpes viruses can also lead to the generation of dsRNA structures during their replication and thus be recognized by TLR3 [126]. Likewise, TLR7/8 sense ssRNA molecules that are produced by many RNA viruses. Better-known for its ability in binding CpG-containing DNA and genomic DNA originating from bacteria, TLR9 can also detect and become active upon CpG-containing DNA derived from many DNA viruses [126]. Once engaged by their ligands, TLR7/8 and 9 use the adapter protein MyD88 to recruit IRAK family kinases in complexes with the E3 ubiquitin ligases TRAF3 and TRAF6, the protein kinase IKKα, and the IRF7 transcription factor. Within this macromolecular complex, IRF7 is phosphorylated by IKKα and translocates to the nuclear compartment, where it induces the expression of type I IFN [127]. Through its ability to control the activation of the MAPKKK TAK1 and the IKK complex, TRAF6 also positively regulates NF-κB and AP1 transcription factors and thus leads to the initiation of a pro-inflammatory gene program (Figure 5) [128,129] (and reviewed in [130,131,132]). As IKKα is essential for the induction of type I IFN, it is interesting to note that this kinase has also been shown to phosphorylate Ser33 of β-catenin and to help TCF-mediated transcription [133]. Thus, by extension, a possibility exists that GSK-3 plays a role in TLR7/8/9 signaling events, thus leading to a type I IFN antiviral response.

On the other hand, the role of GSK-3β in TLR 9 innate pro/anti-inflammatory cytokine responses was firmly established years ago [110] and has been the subject of many excellent reviews [6,134,135,136]. Briefly, its inactivation, following TLR2/4/5/9-mediated PI3K-AKT activation, has been shown to increase the binding of CREB to CBP but, at the same time, to diminish the ability of CBP to interact with the p65 (RelA) NF-κB subunit, thereby enhancing anti-inflammatory cytokine and reducing pro-inflammatory cytokine production [110,135]. 

**Figure 6:**
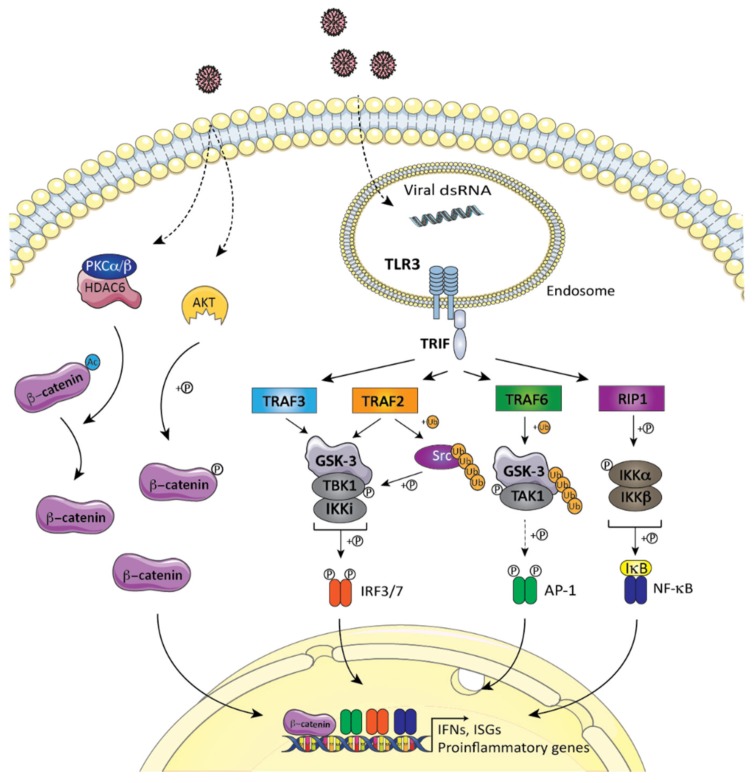
GSK-3/β-catenin in endosomal nucleic acid sensing by TLR3. The engagement of dsRNA molecules with TLR3 triggers activation of TRIF adaptor, which scaffolds the TRAF6-TAK1, RIP1-IKK, and TRAF3-TBK1 complexes, thus leading to the activation of the AP1, NF-κB, and IRF3 transcription factors, respectively, to produce pro-inflammatory cytokines and type I IFN. Catalytically inactive GSK-3β acts as a signaling platform required for TRIF-mediated downstream signaling following TLR3 engagement. GSK3β physically associates with TRAF6 and undergoes TRAF6-dependent K63-polyubiquitination at Lys183, resulting in the formation of the TRIF-TRAF6-TAK1 signaling module and AP1 activation. GSK-3β associates with the TRIF/TRAF3/TBK1 complex and is required for TLR3-mediated TBK1 and IRF3 activation. Moreover, β-catenin mediates the interaction of CBP with IRF3 following Akt-mediated phosphorylation at Ser552 or HDAC6-mediated deacetylation at Lys49. The model was created using Servier Medical Art templates (www.servier.com) licensed under a CC BY 3.0 license (https://creativecommons.org/licenses/by/3.0/).

The possible involvement of GSK-3 in nucleic acid-sensing, TLRs-induced type I IFN immune response has only been verified thus far in TLR3 signaling events. In opposition to TLR7/8/9, this receptor exclusively signals through the TRIF adaptor, which scaffolds the TRAF6-TAK1, RIP1-IKK, and TRAF3-TBK1 complexes, thus leading to the activation of the AP1, NF-κB, and IRF3 transcription factors, respectively [137,138,139,140,141] (Figure 6). Interestingly, GSK-3β, but not GSK-3α, was recently shown to physically associate with TRAF6 following its exposition to the TLR3 poly agonist (I:C), a dsRNA-mimicking agent. Within this complex, GSK-3β undergoes TRAF6-dependent K63-polyubiquitination at Lys183, a modification required for the formation of the TRIF-TRAF6-TAK1 signaling module and AP1 activation [30]. The presence of GSK-3β, but not its catalytic activity, is also required for the production of type I IFNβ. In fact, GSK-3β is found in immunocomplexes with TRIF, TRAF3, and TBK1, and it is required for TLR3-mediated TBK1 and IRF3 activation [30]. Thus, in TLR3 signaling, catalytically inactive GSK-3β acts as a signaling platform required for the TRIF-mediated activation of the TAK1-AP1 and TBK1-IRF3 signaling modules (Figure 6). The involvement of GSK-3β in TLR3-mediated NF-κB activation is still uncertain, as conflicting results have been documented using different loss-of-function approaches [30,75].

The tyrosine kinase Src is also implicated in TLR3-mediated type I IFN antiviral immunity [142], and a model was recently proposed where, when recruited to the nucleic acid-sensing PRR adaptor proteins TRIF, MAVS, and STING, Src acts as a TBK1-activating kinase required for TBK1 transautophosphorylation on Ser172 and IRF3 activation [143]. Interestingly, the TLR3-induced phosphorylation of Src at Tyr416 (and its activation) also requires GSK-3β [144]. In this context, it has been proposed to bridge TRAF2, Src, and TBK1, thus allowing for the TRAF2-dependent K63-polyubiquitination of Src at Lys295, its autophosphorylation at Tyr416, and its TBK1 activation. GSK-3β deficiency significantly reduces the recruitment of TRAF2, Src, and TBK1 to TLR3 but has no effect on TRIF following poly (I:C) stimulation, again positioning the role of GSK-3β downstream of TRIF in TLR3 signaling (Figure 6).

The role of β-catenin in TLR3 signaling resembles the model described above, where, upon poly (I:C) treatment, Akt1 phosphorylates β-catenin on Ser552 [145] and PKCβ-activated HDAC6 enables its deacetylation, thus resulting in its accumulation into the nuclear compartment where it associates with the CBP co-activator, aiding in establishing an IRF3-dependent antiviral gene program [79].

## 7. Conclusions and Future Perspectives

Because of their roles in many physiological conditions, it is reasonable to propose that chronic and systemic inhibition of GSK-3 isoforms with small molecules could lead to significant side-effects. As recently reviewed, isoform-selective inhibitors will definitively become exciting pharmacological tools to test whether GSK-3 isoforms remain viable cellular targets [26]. Concerning the role of GSK-3 in the antiviral type I IFN response, its inhibition with the current drugs that target both isoforms might also not be an ideal scenario, except maybe in the context of septic shocks or autoimmunity involving interferonopathies [146,147]. As for β-catenin, this co-activator is devoid of any enzymatic activity, so its targeting is not trivial. Drug discovery efforts have so far focused on the identification of small-molecule inhibitors of the interaction between β-catenin, TCF transcription factors, and tankyrase inhibitors, with marginal success yet achieved [148]. An alternative therapeutic approach would be to promote the degradation of β-catenin by inhibiting the deubiquitinase (DUB) that controls its turnover [149]. Future DUB inhibitors that selectively decrease the expression level of β-catenin could also find a place in future therapies that aim to treat overt type I IFN responses. Though these are future efforts towards the discoveries of future entities targeting GSK-3 and β-catenin, one of the next fundamental steps will be to address the possible reciprocal roles of the IKK-related kinases over the GSK-3 isoforms, as recently observed for IKKε and GSK-3α [150]. Studies addressing their interactome networks and signaling hubs/clusters that are possibly transient and highly regulated in infected cells (possibly in response to different viruses) will also be challenging but essential. Results from such analysis could open avenues for new areas of intracellular signaling pathway-targeted research in the context of virus infection. As innate immune signaling have potential roles in cancer immune surveillance [151,152] and even in promoting tumor-supporting environments [153], the fine-tuning of the RLR, TLR, and cGAS-STING-dependent cellular responses through the GSK-3/β-catenin axis could also help in the development of future cancer immune therapies. Similarly, since viral infection is still a major cause of cancer, targeting the GSK-3/β-catenin axis in the context of viral oncogenesis could help prevent deleterious outcomes arising from chronic infection to different viruses including EBV, HBV, HCV, and KSHV.
